# Tinea faciei in a newborn due to *Trichophyton tonsurans*

**DOI:** 10.7555/JBR.27.20120102

**Published:** 2012-12-19

**Authors:** Meihua Fu, Yiping Ge, Wei Chen, Suying Feng, Xiaodong She, Xiaofang Li, Weida Liu

**Affiliations:** Department of Mycology, Institute of Dermatology, Chinese Academy of Medical Sciences & Peking Union Medical College, Nanjing, Jiangsu 210042, China

**Keywords:** tinea faciei, *Trichophyton tonsurans*, neonatal

## Abstract

We report here the first case of neonatal tinea faciei caused by *Trichophyton tonsurans* in mainland China. The mother of the infant had tinea corpris and tinea capitis while the father had tinea incongnito. The infections in the parents were mycologically confirmed to be due to *Trichophyton tonsurans*. Ttinea faciei in the infant was cured after two-week topical use of amorolfine cream. The mother ceased breastfeeding and took oral terbinafine for 4 weeks. No recurrence was observed in the infant during 12 months of follow-up.

## INTRODUCTION

*Trichophyton* (*T*.) *tonsurans* is one of the major causative fungi for tinea capitlis in children. The prevalence of this infection has been rising in recent years[1]. It is particularly common in the USA, accounting for 21.1%–44.9% of all dermatophyte infections[2],[3]. Coloe et al.[4]. reported that *T. tonsurans* (88.9%) was the predominant causative agent for tinea capitis in 189 children with a positive scalp culture in Columbus, Ohio, USA. However, there are few cases of tinea capitis in neonates caused by *T. tonsurans*. Up to now, only one case of neonatal ringworm due to this species was reported in the Neonate Intensive Care Unit in Australia and one case of tinea capitis was reported in Korea[5],[6]. No data are available in China. Here, we report the first case of neonatal tinea faciei caused by *T. tonsurans*.

## CASE REPORT

**Fig 1:**
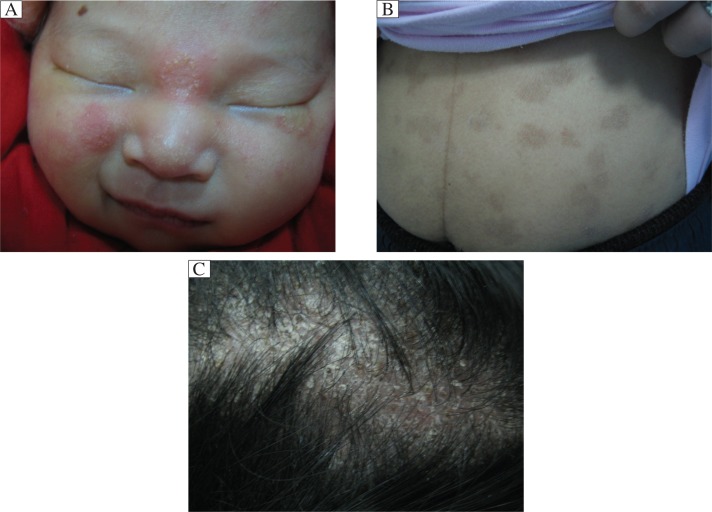
The clinical photos of patients. A: Lesions on the infant's face show round erythema with papules and scales. B: Lesions on the mother's abdomen show red papules with the center resolved and were hyperpigmented. C: Lesions on the mother's scalp show erythema and sticky scales with small pustules, and the appearance of black dots after hair is broken and removed. Use of photographs was permitted by the patients or legal surrogates.

A 15-day old male infant from Lu He of Jiangsu Province came to our clinic due to erythema and papules on the face for two days. Erythema, papules and vesicles appeared from the 13^th^ day, and gradually extended. The infant was delivered at full term and breast-fed. The general condition of the infant was good, and no abnormalities were found. Dermatological examinations showed round erythema with a diameter of 1.5 cm at the nasal root, with papules and scales. On the right cheek and beneath the left eye, there were annular erythema, papules and scales, approximately 1×1 cm^2^ in size. The margin was demarcated with vesicles along the rim. The central lesions were subsided (***Fig. 1A***).

The mother was 27 years old. At the third month of pregnancy, her neck developed itching erythema and scale, but she did not take any medication because of concern of side effects of medication on fetus development. Later, new lesions gradually developed on her back and abdomen. She came to our clinic with the infant. Physical examinations showed multiple irregular lesions on the chest and abdomen with red papules on the rim with the center resolved and hyperpigmented (***Fig. 1B***). On her head, there were erythema and sticky scales with small pustules. The hair broke off on the surface of the scalp, leaving the appearance of a black dot, and was easily removed (***Fig. 1C***). The father was 30 years old and had several red papules and erythema on his abdomen. The lesions were irregular, scaly and centrally resolved.

**Fig 2:**
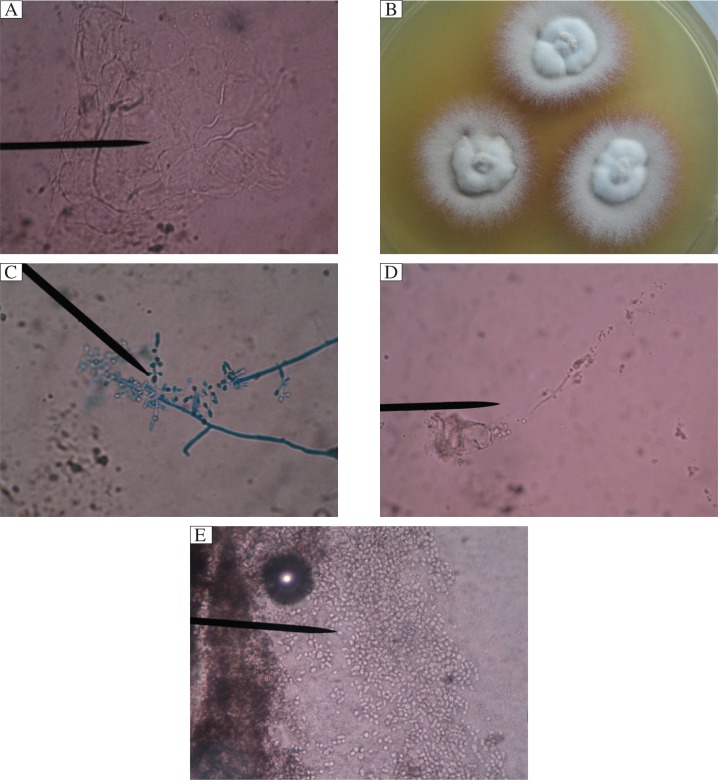
The results of the laboratory examination. A: Hyaline septate hyphae were found by microscopic examination in specimen from the edge of the infant's facial lesions. B: Colony at day 10 on SCAA at 25°C showed that white short fluffy hyphae on the surface and red pigmentation appeared on the edge. C: Microscopic structure of the colony showed that microconidia were produced in abundance and most formed on loosely clustered branches, sessile, clavate, cylindrical or balloon-shaped. D: Abundant branched, septate hyphae was positive by KOH test in the skin samples from the edge of the mother lesions. E: Chain-like spores inside of the hairs were revealed by direct microscopic examination in the hair samples from the mother's head.

Specimens were taken from the edge of the infant's facial lesions with a cotton swap. A potassium hydroxide (KOH) wet mount showed hyaline septate hyphae (***Fig. 2A***). The sample was inoculated on Sabouraud dextrose agar containing 0.5% cycloheximide and 1% chloramphenicol (SCAA) at 25°C. Colonies started to grow at day 5, initially appearing as a grey, flat and powdery colony without red pigment. At day 10, the center of the colony developed white short fluffy hyphae on the surface with red pigmentation on the edge, which became darker within 3 weeks. The reverse was also dark red (***Fig. 2B***). Microscopic examination showed that microconidia were produced in abundance, most forming loosely clustered branches, sessile, clavate, cylindrical or balloon-shaped. Neither macroconidia nor chlamydospore was observed (***Fig. 2C***).

The skin samples were taken from the edge of the mother's lesions. A KOH test showed abundant branched and septate hyphae (***Fig. 2D***). Hair samples were also taken for direct KOH examination, which revealed that chain-like spores were inside of the hairs (***Fig. 2E***). Branched septate hyphae were also detected in the scales from the head. All the samples were cultured on SCAA as described above and the pathogen was identified as *T. tonsurans*. Skin samples taken from the edge of the father's lesions on the abdomen has a negative result on microscopic examination, but the SCAA culture was positive and revealed *T. tonsurans* infection.

The infant was given topical 1% amorolfine cream. The lesions were resolved in two weeks. The infant's mother was also given 1% amorolfine cream for topical use on her abdomen. The lesions were cured in two weeks. Meanwhile, a 2% ketoconazole shampoo was prescribed for her hair washing. One month later, her hairs and scales were still positive by microscopic examination. Six months after delivery, the mother was given oral terbinafine 250 mg/d for 4 weeks, and all lesions were cured after three months. The father's lesions disappeared after topical use of 1% amorolfine cream for two weeks. No relapse from any family member was observed during one year follow-up.

## DISCUSSION

*T. tonsurans* is an anthropophilic dermatophyte, mainly causing black-dot ringworm, tinea kerion and tinea corpris. Human to human transmission is an important way for this fungus infection. The transmission among family members and outbreaks were often noted[1]. There were reports of outbreaks among Japanese judo and wrestling athletes, and an outbreak of hypertrophic tinea corpris caused by *T. tonsurans* in a French high level judo team[7]-[9]. The authors considered that it was mainly associated with direct skin touch and injury[9]. A 2-year prospective, longitudinal study evaluated all preschool-aged children attending a single child care center in Kansas City of United States. A total of 446 children participated in this investigation over the 24-month study period, with the number of participants ranging from 106 to 174 per month. A total of 3,541 scalp cultures were collected, and 1,390 were positive. Among them, *T. tonsurans* accounted for 13.7%–43.8%[10]. An annual 10-year epidemiological survey of pathogenic fungi of inpatients or outpatients including more than 41 units from 25 provinces of China in 1986 and 1996 revealed a prevalence of 1.7% and 0.8%, respectively[11]. In a retrospective study to evaluate tinea captis of Shanghai conducted between 1993 and 2002, *T. tonsurans* was found to be the third most common pathogen, accounting for 9.04% of all isolates[12]. The neonatal tinea faciei in our patient shared the same pathogenic fungus causing concurrent tinea captis and corpris with his parents, illustrating the epidemiological association between close contact and infection. The contact between the infant's face and his mother's breast during breastfeeding may be the main reason for the infection.

Tinea faciei infection is common in children. However, it is extremely rarely seen among infants. Up to the present, *T. rubrum, T. violaceum, Microsporum gypseum, M. canis* and *T. tonsurans* have been reported to cause tinea faciei and tinea copris among neonates aged 2 to 28 days[13]-[18]. Raimer et al.[19] reported three cases of tinea faciei caused by *T. tonsurans* in children. Two children were negative by direct examination but positive by culture. The third child was positive both by direct examination and culture. Meanwhile, *T. tonsurans* was isolated from the scalp of two infants' parents. Ravenscroft et al[20] reported a Caucasian family of two veterinary practitioners and their two children, aged 2 years and 6 months, simultaneously infected with *T. tonsurans*, causing tinea capitis and tinea corporis in the children and tinea corporis in the parents. The parents and the older child were successfully treated with oral terbinafine. The infant was treated with topical terbinafine and ketoconazole shampoo but presented with recurrent tinea capitis for 12 months. Then, he received oral terbinafine, resulting in clinical and mycologic cure. After a further 12 months follow-up, there has been no mycologic evidence of recurrence in any family member.

In the present report, tinea faciei of the infant and tinea corpris of the parents was clinically cured with a two-week topical use of 1% amorolfine cream. Tinea captis in the mother was clinically cured with oral terbinafine for 4 weeks. No signs of recurrence was seen after a follow-up for one year. Tinea is not a common skin disease for neonates and infants, which may be a clue that their parents or other intimate contacts have encountered similar dermatophytosis. Therefore, a complete examination should be carried out, and timely treatment should be applied to prevent the disease from spreading.
